# Dry Eye Disease Among Mongolian and Han Older Adults in Grasslands of Northern China: Prevalence, Associated Factors, and Vision-Related Quality of Life

**DOI:** 10.3389/fmed.2021.788545

**Published:** 2021-11-25

**Authors:** Jianhua Wu, Xiaomei Wu, Han Zhang, Xiaoguang Zhang, Jie Zhang, Yanqiu Liu, Jun Liu, Lu Lu, Song Zhang, Guisen Zhang, Lei Liu

**Affiliations:** ^1^Inner Mongolia Chaoju Eye Hospital, Inner Mongolia Chaoju Institute of Eye Disease Control, Hohhot, China; ^2^Department of Clinical Epidemiology and Center of Evidence-Based Medicine, The First Hospital of China Medical University, Shenyang, China; ^3^School of Public Health, Weifang Medical University, Weifang, China; ^4^Tobacco Control, Chinese Center for Disease Control and Prevention, Beijing, China; ^5^Department of Ophthalmology, Anshan Central Hospital, Anshan, China; ^6^Department of Ophthalmology, Changzhi People's Hospital, Changzhi, China; ^7^Department of Ophthalmology, The Fourth Affiliated Hospital of China Medical University, Shenyang, China; ^8^The First Hospital of China Medical University, Shenyang, China; ^9^Department of Ophthalmology, Guangdong Eye Institute, Guangdong Provincial People's Hospital, Guangdong Academy of Medical Sciences, Guangzhou, China; ^10^School of Medicine, South China University of Technology, Guangzhou, China; ^11^Department of Ophthalmology, The First Affiliated Hospital of China Medical University, Shenyang, China

**Keywords:** dry eye disease, prevalence, associated factors, vision-related quality of life, epidemiology—analytic (risk factors)

## Abstract

**Purpose:** Dry eye disease (DED) is projected to have increasing public health burden in China with the aging population. No published studies on the epidemiology of DED have been found in grasslands. We estimated DED prevalence among older adults living in grasslands of northern China and investigated its associated factors and impact on vision-related quality of life (VR-QoL).

**Methods:** A multistage cluster random sampling technique was used to select Mongolian and Han participants aged over 40 from November 2020 to May 2021 in this area. An assessment of DED was performed with Ocular Surface Disease Index (OSDI) questionnaire, Schirmer's I test (ST), and Tear film break up time (TBUT). All the participants completed the Chinese version of National Eye Institute Visual Function Questionnaire (NEI-VFQ-25) assessing VR-QoL.

**Results:** Of the 1,400 enumerated residents, 1,287 were examined. The overall age and gender standardized prevalence of DED was 34.5%, of which, 32.6% of Mongolian and 35.4% of Han had DED. In a multivariate model, statistically significant associations were found with advancing age [odds ratio (OR) 1.03, 95% confidence interval (CI) 1.02–1.04], female gender (OR 1.32, 95% CI 1.04–1.68), smoking (OR 0.7, 95% CI 0.5–0.98), anti-fatigue eye-drop use (OR 0.56, 95% CI 0.41–0.77), milk product intake (OR 0.55, 95% CI 0.39–0.77), number of household members (OR 0.8, 95% CI 0.72–0.88). DED was associated with lower scores on VR-QoL (β= −0.14, *P* < 0.01). Similar results were observed when analyses were stratified by ethnicity.

**Conclusions:** The novelty-associated factors for DED in the grasslands area were anti-fatigue eye drop use, milk product intake, and number of household members. DED and its components were associated with VR-QoL. Further prospective studies are needed to confirm these findings.

## Introduction

Dry eye disease (DED) is an age-related degenerative condition. It is one of the leading reasons for patients seeking eye care, with prevalence estimates ranging from ~5–50% in population-based studies, depending on population studied and diagnostic criteria (1). According to geographic (i.e., high latitude), climatic (i.e., humidity levels), and environmental variations (i.e., ultraviolet radiation) associated with DED, it is more common in Asian populations (2–5). In China, a previous meta-analysis has shown that the pooled prevalence of dry eye syndrome (DES) is 17%, and that subjects living in the Northern and Western China have significantly higher prevalence rates than those living in other areas (6).

The area of grasslands is about 3.84 million square kilometers. The grasslands in northern of China are mainly located in the Inner Mongolia Autonomous Region, and have high latitude, low humidity, increased ultraviolet (UV) light, and windy conditions. Most of the residents living in these grasslands area are herders (Mongolian and Han). Because of their unique living and eating habits (more milk and meat), which lead to differences in diseases, it is very important to understand the eye health of residents in grasslands area.

In 2006, Guo et al. conducted an epidemiology investigation of DED among Chinese Mongolian in Henan county, Qinghai province of China, which showed that the crude prevalence of symptomatic dry eye measured with a six-item validated questionnaire was 50.1%, and that its independent factors included increased age, presence of age-related cataract, and pterygium (7). In addition, to the best of our knowledge, limited data are available on the prevalence and related factors of DED in grasslands area.

Recently, many reports have suggested that DED can affect vision-related quality of life (VR-QoL) (1, 8), but there were only a few population-based studies on the association between DED and VR-QoL worldwide (9–13). Furthermore, the impact of DED on VR-QoL in grasslands populations is relatively unknown.

The aims of this investigation in the grasslands area, which is predominately composed of Chinese Mongolian and Han older adults aged over 40 years, were to determine the prevalence of DED, identify independent associated factors, and quantify their impact on VR-QoL.

## Methods

### Study Introduction

This grasslands multiethnic eye disease epidemiological study is a cross-sectional, population-based one in eastern, middle, and western parts of the Inner Mongolia Autonomous Region and Ningxia province, China. This investigation is divided into three parts. The first stage was conducted in the middle grasslands area, and focused on Mongolian and Han ethnicities. The second stage will be conducted in eastern the grasslands area, and will focus on Ewenki, Oroqen, and Daur ethnicities. The third stage will be conducted in the western grasslands area, and will focus on Hui ethnicity. This study is the first stage.

### Study Area

Multistage cluster sampling was performed to select two areas (Xilingol and Ulanqab) from the grasslands area located in the northern and middle parts of the Inner Mongolia Autonomous Region, China (Supplementary Figure 1). The three areas (Ujimqin Banner, Sonid Banner, and Siziwang Banner) were chosen by primary cluster sampling, and then relevant county seats were randomly selected by secondary cluster sampling. The county seats were divided into two levels (urban and rural) according to economic conditions. The study area runs from 111° 68 to 117° 58 E in longitude and 41°37 to 44°60N in latitude. The mean annual temperature ranges from 1 to 6°C in 2019. The region is far away from the ocean and resides in a low-humidity wide area. The mean annual precipitation range is between 170 and 350 mm, with 60 −90% falling during the growing season from April to August.

### Study Population

According to the data obtained from the 2010 nationwide population census, there are ~0.46 million with two predominant ethnic groups, Mongolian and Han, in the study region. An estimated prevalence of DED of 31.4% was made reference to our sample (2). The formula: *n* = *Z*^2^*p*(1–*p*)/*q*^2^ was used to calculate sample size, whereas *Z* = 1.96, *p* = 0.314, *q* = 0.1 *p*. The estimated minimum sample size was 839. We added an additional 15% to the minimum sample size factoring in possible non-compliance rate and targeted 1,258 subjects. The study sample was stratified to include proportions of Mongolian and Han ethnic groups in two grasslands. All the participants gave informed consent. This study was approved by the ethics committee of Inner Mongolia Chaoju Eye Hospital and adhered to the tenets of the Declaration of Helsinki. This study was registered in Chinese Clinical Trial Registry with No. ChiCTR2000040141.

This study was conducted from November 2020 to May 2021. Only Mongolian and Han subjects aged 40 and above were interviewed for the study. Patients who underwent refractive surgery at latest 3 months and those with an active ocular surface disease were excluded.

### The Questionnaire

In order to acquire participants-reported questionnaires, Chinese version of the Ocular Surface Disease Index (OSDI) and National Eye Institute Visual Functioning Questionnaire-25 (NEI-VFQ-25) questionnaire were interviewed by well-trained investigators. Previous studies have revealed that these two Chinese version questionnaires were easily administered, socio-culturally acceptable, and understandable (14, 15). The OSDI questionnaire consists of 12 questions, and each question is graded from 0 (indicating no problem) to 5 (indicating a significant problem). OSDI scores were calculated with the formula (sum of scores) × 25/(12 questions). The NEI-VFQ-25 questionnaire includes 12 subcategories: (i) general health; (ii) general vision; (iii) ocular pain; (iv) near vision; (v) distance vision; (vi) social functioning; (vii) mental health; (viii) role difficulties; (ix) dependency; (x) driving; (xi) color vision; and (xii) peripheral vision. The NEI-VFQ-25 questionnaire was scored by the researcher according to the scoring manual. Its score ranges from 0 to 100, and designates worst state to normal visual function, respectively. Furthermore, socio-demographic characteristics (such as cultural level, family income, and employment), lifestyle, screen exposure, and dietary supplements, as well as medical history were acquired from each participant. Milk product intake was categorized as occasional (<1 time/day) or regular (more than one time/day). Anti-fatigue eye drop was defined as topical medications improving patient comfort, such as eye lubricants and artificial tear drops.

#### Examination

##### Tear Film Break Up Time

One drop of topical anesthesia, 0.4% oxybuprocaine hydrochloride ophthalmic solution (Benoxil; Oxybuprocaine, Santen, Japan), was instilled. After 1 min, the subjects were instructed to look up to apply a fluorescein sodium ophthalmic strip into the inferior fornix. Each subject should blink three times, then keep their eyes open as long as possible. The time between the last blink and the first dry spot around the central cornea was recorded under cobalt blue light. The average of three measurements was recorded as the final TBUT.

##### Schirmer's I Test

Schirmer's I test is a basic tear secretion test without anesthesia and performed with tear strip (30 mm; Jingming Tianjin, China). The tear strip was placed in the mid-lateral portion of the lower fornix, and the subjects were instructed to close their eyes for 5 min. After that, the length of the wetting strip was recorded as the level of tear secretion.

### Definitions

DED in this study was defined as OSDI scores of 13 and above plus one of the items as shown below: (i) Schirmer's I test reading value less than 10 mm (ii) TBUT value less than 10 s.

### Statistical Analysis

All data were entered into an Excel form. The SPSS 23.0 (IBM Corp., Armonk, NY, United States) statistical software was applied to analyze the data. For qualitative indicators, we used frequency and percentage for statistical description, and Wilcoxon rank sum test was performed for analysis. The normal distribution continuous data were expressed by the mean ± standard deviation (mean ± SD), and then Students' *t*-test was performed to determine if there were significant differences between groups. According to the sixth national census of 2010, the prevalence of DED was standardized by age and gender composition data of 40 years and above (http://www.stats.gov.cn/tjsj/pcsj/rkpc/6rp/indexch.htm). After adjusting for age, gender, and other variables, a multivariate logistic regression model was used to estimate the odds ratio (OR) and 95% confidence interval (CI) of DED factors. A Pearson's linear correlation analysis was performed to analyze the relationship between NEI-VFQ-25 scores and OSDI, TBUT as well as Schirmer's I test variables. Multiple linear regression was used, and the VR-QoL subscales were used as dependent variables. DED clinical indicators were used as independent variables and inputted into the multiple linear regression model to detect their impacts on VR-QoL. The test standard is α = 0.05 (bilateral), and a *P*-value of <0.05 was considered statistically significant.

## Results

Totally, there were 1,400 participants eligible for this survey. Forty-four subjects declined to participate. Fifty-six subjects were excluded from the study, of which 5 underwent refractive surgery within at latest 3 months, and 51 had an active ocular surface disease. Thirteen subjects were eliminated because of missing data. Finally, there were 1,287 people (age 61.24 ± 9.54 years, 66.4% women) who actually participated in the ophthalmology and medical examinations, and the response rate was 91.93%. There were 816 Mongolian (age 60.46 ± 9.29 years, 64.1% women) and 471 Han (aging 62.59 ± 9.82 years, 70.3% women) participants. Among all the participants, the number of current smokers, never-smokers, and ex-smokers was 198, 1,053, and 37, respectively.

### Prevalence

**Figure 1:**
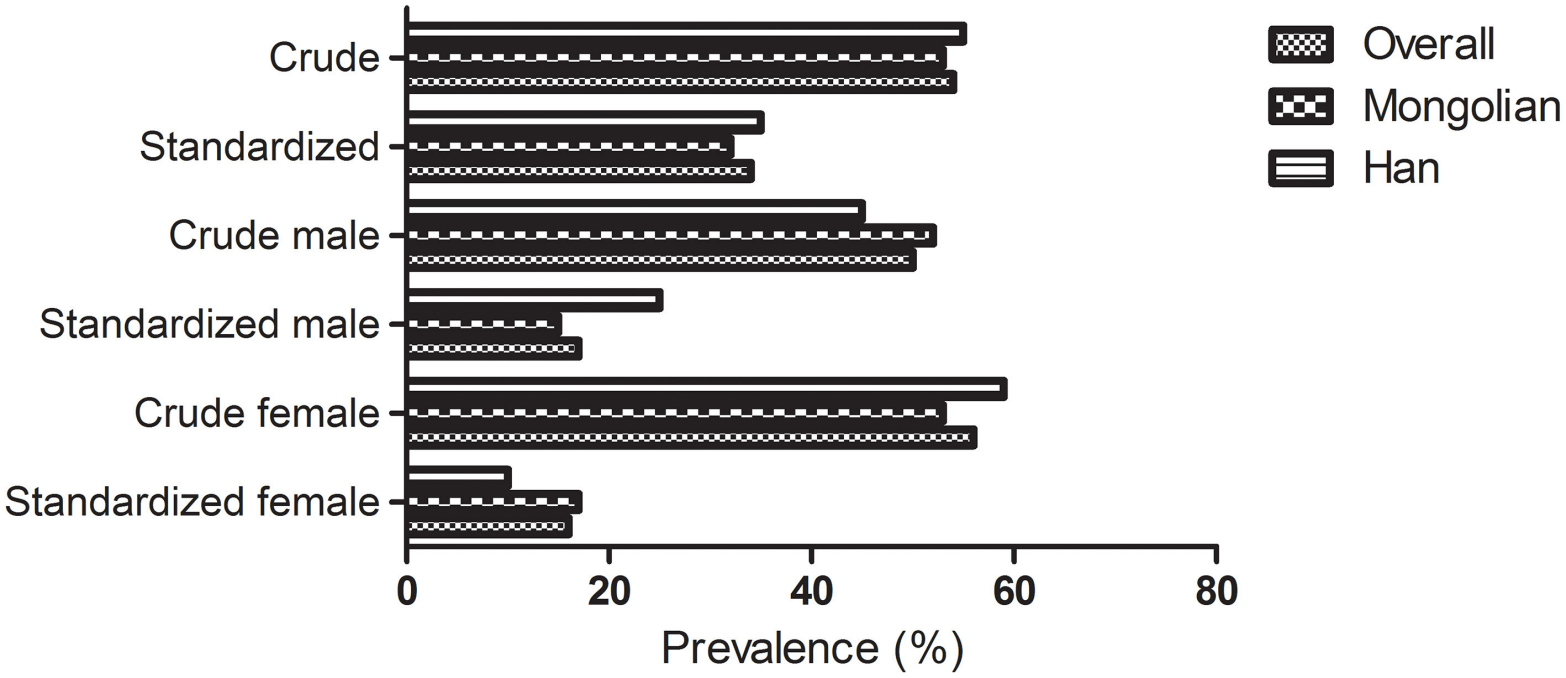
Crude and standardized prevalence of dry eye disease stratified by gender and ethnicity in the grasslands.

A total of 696 participants fulfilled the diagnostic criteria for DED, with a crude prevalence rate of 54.2%, and the crude prevalence of DED in Mongolian and Han ethnicities was 53.6 and 55.1%, respectively. Based on the 2010 sixth National Census of China, the overall age and gender standardized prevalence rate of DED was 34.5% in the grasslands, and the age- and gender-standardized prevalence of DED in Mongolian and Han ethnicities was 32.6 and 35.4%, respectively. The prevalence of DED is shown in Figure 1.

### Factors Associated With the Presence of DED

According to the univariate analysis (Table 1), participants with DED were more likely to be older, of female gender, shorter in height, and with anti-fatigue eye-drop use, occasional milk intake, less number of household members, less annual household incomes, and lower Schirmer's I test and TBUT but higher OSDI scores (all *P* < 0.05). Unadjusted univariate differences in DED among the Han and Mongolian participants by demographic, lifestyle and other factors are presented in Supplementary Tables 1, 2. The multivariate logistic regression demonstrated that advancing age, female gender, no smoke, anti-fatigue eye-drop use, milk product intake, number of household members, and Schirmer's I test, TBUT, and OSDI scores were independently associated with DED (all *P* < 0.05, Table 2). Similar factors were found in the ethnic-specific analysis (Supplementary Tables 3, 4). However, female gender and milk product intake were not associated with DED among the Chinese Mongolians.

**Table 1:** Characteristics of the participants.

	**N/Mean ± sd**	**Range**	**DED**	**No DED**	***P***
Total	1,287	–	697 (54.2%)	590 (45.8%)	
**Ethnic**					
Mongolian	816	–	437 (53.6%)	379 (46.4%)	0.57
Han	471	–	60 (55.2%)	211 (44.8%)	
**Residence**					
Rural	404	–	223 (55.2%)	181 (44.8%)	0.61
Urban	883	–	474 (53.7%)	409 (46.3%)	
**Gender**					
Male	433	–	211 (50.1%)	216 (49.9%)	**0.03**
Female	854	–	481 (56.3%)	373 (43.7%)	
**Occupation**					
Worker	22	–	10 (45.5%)	12 (54.5%)	0.86
Farmer	490	–	268 (54.7%)	222 (45.3%)	
Staff	60	–	32 (53.3%)	28 (46.7%)	
Other	715	–	386 (54.1%)	329 (45.9%)	
**Smoke**					
Current	198	–	111 (56.1%)	87 (43.9%)	0.28
Never	1,053	–	563 (53.5%)	490 (46.5%)	
Former	37	–	23 (62.2%)	14 (37.8%)	
**Drink**					
Current	116	–	57 (49.2%)	59 (50.8%)	0.55
Never	1,135	–	617 (54.4%)	518 (45.6%)	
Former	36	–	20 (55.6%)	16 (44.4%)	
**Level of education**					
Primary school	689	–	397 (57.6%)	292 (42.4%)	0.11
Junior high school	318	–	162 (50.9%)	156 (49.1%)	
Senior high school	158	–	80 (50.6%)	78 (49.4%)	
College	120	–	57 (47.5%)	63 (52.5%)	
Unclear	2	–	1 (50%)	1 (50%)	
**Diabetes**					
With	137	–	72 (52.6%)	65 (47.4%)	0.70
Without	1,088	–	587 (54%)	501 (46%)	
Unclear	62	–	35 (56.5%)	27 (43.5%)	
**Hypertension**					
With	474	–	276 (58.2%)	198 (41.8%)	0.08
Without	768	–	397 (51.7%)	371 (48.3%)	
Unclear	45	–	24 (53.4%)	21 (46.6%)	
Anti-fatigue eye-drop use
Yes	204	–	132 (64.7%)	72 (35.3%)	**<0.01**
No	1,083	–	564 (52.1%)	519 (47.9%)	
**Milk products intake**					
Regular	1,028	–	576 (56.1%)	452 (43.9%)	**0.01**
Occasional	259	–	122 (47.2%)	137 (52.8%)	
Age (years)	61.24 ± 9.54	(22–93)	62.71 ± 9.07	59.51 ± 9.80	**<0.01**
Screen exposure per day (*h*)	2.65 ± 1.81	(0–12)	2.61 ± 1.84	2.69 ± 1.77	0.39
Number of household members (*n*)	2.58 ± 1.21	(1–11)	2.4 ± 1.19	2.79 ± 1.19	**<0.01**
Annual household incomes (per 10,000 Yuan)	4.07 ± 4.83	(0–60)	3.77 ± 3.91	4.42 ± 5.71	**0.02**
Height (cm)	160.42 ± 8.43	(103–188)	159.57 ± 8.41	161.44 ± 8.35	**<0.01**
Weight (kg)	70.64 ± 14.37	(41–170)	70.22 ± 14.75	71.13 ± 13.90	0.26
BMI	27.35 ± 5.42	(14.88-66.92)	27.56 ± 5.33	27.24 ± 5.17	0.27
Height (cm)	98.25 ± 28.58	(39–171)	99.1 ± 36.78	97.24 ± 13.44	0.25
Waist (cm)	86.69 ± 12.63	(15–140)	86.94 ± 12.34	86.4 ± 12.97	0.44
SBP (mmHg)	139.75 ± 24.72	(85–200)	140.2 ± 28.96	139.22 ± 18.52	0.48
DBP (mmHg)	82.22 ± 14.19	(50–144)	81.91 ± 14.06	82.58 ± 14.35	0.40
Hart rate (per minute)	81.38 ± 13.69	(42–122)	81.27 ± 14.01	81.51 ± 13.31	0.76
Schirmer's I test (mm)	8.55 ± 7.34	(0–30)	6.23 ± 5.75	11.28 ± 8.05	**<0.01**
TBUT (s)	5.98 ± 3.99	(0–15)	3.86 ± 2.50	8.48 ± 3.97	**<0.01**
OSDI score	21.82 ± 13.00	(0–100)	26.82 ± 10.99	15.91 ± 12.71	**<0.01**

*DED, dry eye disease; BMI, body mass index; SBP, systolic blood pressure; DBP, diastolic blood pressure; TBUT, Tear film break up time; OSDI, ocular surface disease index; SD, standard deviation. Bold values mean statistically significant*.

**Table 2:** Logistic regression analysis of risk factors associated with definite DED.

	**Crude**			Adjusted^*^			Adjusted^**^		
	**OR**	**95% CI**	***P***	**OR**	**95% CI**	***P***	**OR**	**95% CI**	***P***
Age (years)	1.04	1.02–1.05	**<0.01**	1.04	1.03–1.05	**<0.01**	1.03	1.02–1.04	**<0.01**
Gender (female)	1.29	1.02–1.62	**0.03**	1.29	1.02–1.64	**0.03**	1.32	1.04–1.68	**0.02**
Ethnic (Mongolian)	0.94	0.75–1.18	0.57	1.03	0.82–1.30	0.79	1.06	0.83–1.34	0.66
Residence (rural)	1.31	0.74–1.19	0.20	0.99	0.78–1.26	0.92	0.92	0.69–1.21	0.53
**Occupation**									
Worker	Ref			Ref			Ref		
Farmer	1.45	0.61–3.42	0.40	1.31	0.55–3.11	0.55	1.12	0.46–2.71	0.80
Staff	1.37	0.51–3.66	0.53	1.32	0.49–3.57	0.58	1.18	0.44–3.22	0.74
Other	1.41	0.60–3.31	0.43	1.33	0.56–3.15	0.52	1.11	0.46–2.66	0.82
**Smoke**									
Current	Ref			Ref			Ref		
Never	0.90	0.66–1.22	0.50	0.70	0.50–0.98	0.04	0.70	0.50–0.98	**0.04**
Former	1.57	0.72–3.41	0.26	1.33	0.60–2.95	0.48	1.36	0.61–3.00	0.45
**Drink**									
Current	Ref			Ref			Ref		
Never	1.23	0.84–1.81	0.29	0.92	0.60–1.40	0.68	0.92	0.60–1.41	0.70
Former	1.31	0.61–2.83	0.49	1.28	0.58–2.82	0.55	1.25	0.56–2.77	0.58
Screen exposure per day (*h*)	0.97	0.92–1.03	0.39	1.04	0.98–1.12	0.20	1.04	0.97–1.11	0.27
Anti-fatigue eye-drop use (no)	0.58	0.42–0.80	**<0.01**	1.04	1.03–1.05	**<0.01**	0.56	0.41–0.77	**<0.01**
Milk products intake (regular)	0.69	0.53–0.91	**0.01**	0.64	0.48–0.85	**<0.01**	0.55	0.39–0.77	**<0.01**
**Level of education**									
Primary school	Ref			Ref			Ref		
Junior high school	0.76	0.59–1.00	0.05	0.87	0.66–1.14	0.31	0.91	0.68–1.20	0.49
Senior high school	0.75	0.53–1.07	0.11	0.92	0.65–1.32	0.66	0.93	0.64–1.33	0.68
College	0.67	0.41–0.98	0.04	0.90	0.60–1.36	0.63	0.91	0.61–1.38	0.66
Unclear	0.74	0.05–11.81	0.83	0.81	0.05–13.27	0.88	0.69	0.04–11.28	0.80
Number of household members (n)	0.76	0.69–0.83	**<0.01**	0.80	0.72–0.88	**<0.01**	0.80	0.72–0.88	**<0.01**
Annual household incomes (per ten thousand yuan)	0.97	0.95–1.00	0.02	0.99	0.97–1.02	0.51	1.00	0.98–1.03	0.99
**Diabetes**									
Yes	Ref			Ref			Ref		
No	1.06	0.74–1.51	0.76	1.14	0.79–1.64	0.48	1.15	0.80–1.67	0.46
Unclear	1.31	0.69–2.49	0.40	1.42	0.74–2.74	0.29	1.36	0.70–2.64	0.36
**Hypertension**									
Yes	Ref			Ref			Ref		
No	0.77	0.61–0.97	0.03	0.90	0.71–1.14	0.38	0.93	0.73–1.18	0.54
Unclear	0.83	0.44–1.54	0.55	0.86	0.46–1.63	0.65	0.85	0.45–1.63	0.63
Height (cm)	0.97	0.96–0.99	<0.01	0.99	0.97–1.00	0.13	0.99	0.97–1.01	0.19
Weight (kg)	1.00	0.99–1.00	0.26	1.00	0.99–1.01	0.83	1.00	0.99–1.01	0.98
BMI	1.01	0.99–1.03	0.21	1.01	0.99–1.03	0.50	1.01	0.99–1.03	0.43
Height (cm)	1.00	1.00–1.01	0.31	1.00	1.00–1.01	0.35	1.00	1.00–1.01	0.37
Waist (cm)	1.00	1.00–1.01	0.44	1.00	0.99–1.01	0.63	1.00	0.99–1.01	0.66
SBP (mmHg)	1.00	1.00–1.01	0.49	1.00	0.99–1.00	0.57	1.00	0.99–1.00	0.61
DBP (mmHg)	1.00	0.99–1.00	0.40	1.00	0.99–1.01	0.41	1.00	0.99–1.00	0.38
Hart rate (per minute)	1.00	0.99–1.01	0.76	1.00	0.99–1.01	0.43	1.00	0.99–1.01	0.38
Schirmer's I test (mm)	0.90	0.88–0.92	**<0.01**	0.90	0.88–0.92	**<0.01**	0.90	0.88–0.92	**<0.01**
TBUT (s)	0.70	0.67–0.72	**<0.01**	0.70	0.67–0.73	**<0.01**	0.69	0.67–0.72	**<0.01**
OSDI score	1.10	1.09–1.12	**<0.01**	1.10	1.08–1.11	**<0.01**	1.10	1.08–1.11	**<0.01**

*DED, dry eye disease; BMI, body mass index; SBP, systolic blood pressure; DBP, diastolic blood pressure; TBUT, Tear film break up time; OSDI, ocular surface disease index; OR, odds ratio; CI, confidence interval*.

* *Adjusted with age, gender, and ethnicity*.

** *Adjusted with age, gender, ethnicity, and number of household members*.

*Bold values mean statistically significant*.

### Vision-Related Quality of Life

The correlation between DED characteristics and VR-QoL in all the participants is shown in Table 3. Schirmer's I test was significantly correlated with the three NEI-VFQ-25 subscales: general health (*r* = −0.06), general vision (*r* = 0.06), and dependency (*r* = 0.06). TBUT was correlated with the NEIVFQ-25 overall scores (*r* = 0.06), as well as with three subscales: general health (*r* = 0.06), general vision (*r* = 0.07), and driving (*r* = 0.1) in all the participants. Furthermore, OSDI score was correlated with the NEI-VFQ-25 overall score (*r* = −0.47) and all its 12 subscales. Schirmer's I test and TBUT were not correlated with quality of life in the Han participants, while OSDI score was significantly correlated with the NEI-VFQ-25 scores (see Supplementary Table 5). Among the Mongolians, Schirmer's I test and TBUT were correlated with general health (*r* = −0.08) and general vision (*r* = 0.08), respectively. Moreover, the OSDI score of the Mongolians was significantly correlated with the all NEI-VFQ-25 scores, except general health (see Supplementary Table 6).

**Table 3:** Correlation between DED subscale and NEI-VFQ-25 subscale.

	**Overall**	**General**	**General**	**Ocular**	**Near**	**Distance**	**Social**	**Mental**	**Role**	**Dependency**	**Driving**	**Color**	**Peripheral**
		**health**	**vision**	**pain**	**activities**	**activities**	**functioning**	**health**	**difficulties**			**vision**	**vision**
Schirmer's I test (mm)	−0.01	−0.06^*^	0.06^*^	−0.03	0.01	0.03	0.02	−0.02	0.02	0.06^*^	−0.02	0.01	−0.00
TBUT (s)	0.06^*^	0.06^*^	0.07^*^	−0.03	0.02	0.04	0.03	−0.01	0.05	0.01	0.10^*^	0.01	0.03
OSDI score	−0.47^**^	−0.15^**^	−0.40^**^	0.10^**^	−0.09^**^	−0.26^**^	−0.24^**^	−0.09^**^	−0.22^**^	−0.48^**^	−0.55^**^	−0.12^**^	−0.21^**^

*DED, Dry eye disease; TBUT, Tear film break up time; OSDI, ocular surface disease index; NEI-VFQ-25, National Eye Institute Visual Functioning Questionnaire-25*.

* *P < 0.05*;

** *P < 0.01*.

The results of multiple linear regression for the overall NEI-VFQ-25 score and subscales are presented in Table 4. DED was a predictor for the overall scores (β = −0.14, *P* < 0.01), and general health (β = −0.06, *P* = 0.03), general vision (β = −0.18, *P* < 0.01), ocular pain (β = −0.02, *P* = 0.49), near activities (β = −0.07, *P* = 0.01), distance activities (β = −0.09, *P* < 0.01), and social functioning (β = −0.07, *P* = 0.02). OSDI score was associated with the overall NEI-VFQ-25 score and all the subscales. Schirmer's I test and TBUT were not associated with NEI-VFQ-25 scores. NEI-VFQ-25 and its subscales are used as dependent variables, and demographic and clinical characteristics are used as predictors to be added to the stepwise multiple linear regression model to screen risk factors. Stratification by ethnicity showed similar results (see Supplementary Tables 7, 8).

**Table 4:** Multiple linear regression on dry eye disease and vision-related quality of life based on NEI-VFQ-25 in all the participants.

	**DED**				**OSDI score**				**Schirmer's I test (mm)**				**TBUT (s)**			
			**95%CI**				**95%CI**				**95%CI**				**95%CI**	
	***β*** ^*^	***P***	**Low**	**Up**	β^*^	***P***	**Low**	**Up**	β^*^	***P***	**Low**	**Up**	β^*^	***P***	**Low**	**Up**
Overall	−0.14	**<0.01**	−0.19	−0.09	−0.50	**<0.01**	−0.54	−0.45	0.01	0.69	−0.04	0.06	0.02	0.37	−0.03	0.08
General health	−0.06	**0.03**	−0.11	−0.01	−0.11	**<0.01**	−0.16	−0.06	−0.05	0.06	−0.10	0.01	0.03	0.32	−0.03	0.08
General vision	−0.18	**<0.01**	−0.24	−0.13	−0.39	**<0.01**	−0.44	−0.34	0.04	0.13	−0.01	0.09	0.05	0.06	<0.01	0.10
Ocular pain	−0.02	**0.49**	−0.08	0.04	0.16	**<0.01**	0.11	0.22	0.01	0.88	−0.06	0.05	0.01	0.67	−0.04	0.07
Near activities	−0.07	**0.01**	−0.12	−0.02	−0.24	**<0.01**	−0.29	−0.18	0.01	0.61	−0.04	0.07	0.01	0.95	−0.06	0.05
Distance activities	−0.09	**<0.01**	−0.14	−0.03	−0.29	**<0.01**	−0.34	−0.24	0.02	0.38	−0.03	0.08	0.02	0.56	−0.04	0.07
Social functioning	−0.07	**0.02**	−0.12	−0.01	−0.32	**<0.01**	−0.37	−0.26	−0.01	0.80	−0.06	0.05	0.02	0.56	−0.04	0.07
Mental health	0.05	0.11	−0.01	0.10	−0.14	**<0.01**	−0.19	−0.08	−0.02	0.44	−0.08	0.03	−0.04	0.20	−0.09	0.02
Role difficulties	−0.05	0.06	−0.11	<0.01	−0.24	**<0.01**	−0.29	−0.18	0.02	0.49	−0.04	0.07	0.02	0.60	−0.04	0.07
Dependency	−0.13	**<0.01**	−0.18	−0.07	−0.45	**<0.01**	−0.50	−0.40	0.05	0.08	−0.01	0.10	0.01	0.81	−0.05	0.06
Driving	−0.10	**0.02**	−0.18	−0.02	−0.21	**<0.01**	−0.28	−0.15	−0.02	0.63	−0.10	0.06	0.01	0.98	−0.08	0.08
Color vision	−0.01	0.70	−0.07	0.05	−0.21	**<0.01**	−0.27	−0.15	0.02	0.52	−0.04	0.07	0.01	0.80	−0.05	0.06
Peripheral vision	−0.04	0.19	−0.09	0.02	−0.26	**<0.01**	−0.31	−0.20	−0.03	0.29	−0.09	0.03	0.01	0.94	−0.05	0.06

*TBUT, Tear film break up time; OSDI, ocular surface disease index; CI, confidence interval; DED, dry eye disease; NEI-VFQ-25, 25-item National Eye Institute Visual Functioning Questionnaire*.

* *Adjusted with age, gender, annual household incomes, and diabetes*.

*Bold values mean statistically significant*.

## Discussion

Documented epidemic data on DED among residents in the grasslands area are limited, and, this study sought to determine the prevalence, risk factors of DED, and its role in VR-QoL between the two main ethnicities (Mongolian and Han) in the northern grasslands area in China. The overall age- and gender-standardized prevalence of DED was 34.5%, and, the standardized prevalence in the Mongolian and Han were 32.6 and 35.4%, respectively. In addition, DED was associated with several factors, such as being female and older, smoking, anti-fatigue eye-drop use, regular milk product intake, number of household members, and Schirmer's I test, TBUT, and OSDI scores. Notably, there was a significant correlation between DED components (OSDI, Schirmer's I test, and TBUT) and NEI-VFQ-25 scores. After the multivariate regression analysis, DED and OSDI have significant impacts on VR-QoL among population living in the grasslands.

The prevalence of DED in this study was 54.2%, which is over the range of previous population-based findings (5–50%) (7, 16–20). The participants were relatively older, at 61.24 ± 9.54 years old and with predominance of women (66.4%). However, the age- and gender-standardized prevalence was still higher than that in previous meta-analysis (2). This discrepancy may be due to the special geographical (high latitude) and environmental factors (dust, sand, and drought) as well as the lifestyle of people living in the grasslands area of Northern China. In addition, different techniques used to diagnose DED may also have an impact on this discrepancy. There may be discordance between dry eye signs and symptoms, with the signs being more prevalent and variable than the symptoms (1). Furthermore, there was no significant difference in the prevalence of DED between Mongolian and Han participants in our study.

So far, advancing age, female gender, and smoking are the most common factors associated with DED (21), which were consistent with our findings. However, another population-based investigation in Dubai showed that daily screen time (> 6 h) was positively associated with dry eyes (22, 23), while no association between daily screen exposure time and DED was found in this study. This discrepancy might be due to the different lifestyle of the study participants. Our participants spent most of their time outdoors and, therefore, had low exposure to screen, which might explain the absence of an association between daily screen time and dry eyes in the current study.

It is interesting to note that anti-fatigue eye-drop use was associated with DED. Eye fatigue is a manifestation of DED (24), and many patients were confirmed to use anti-fatigue eye-drops to relieve the syndrome. Furthermore, regular milk product intake might reduce the risk for presence of DED. Recently, a randomized, double-blind, placebo-controlled, parallel group comparative study revealed that H_2_-producing milk appeared to retard the decline in tear stability and may prevent short fTBUT-type DED by decreasing oxidative stress in the lacrimal functional unit (25). In addition, it would be a good idea to identify the effects of milk product intake on DED prevention or treatment in future studies. When stratified by ethnicity, the association was still significant in the Han participants rather than the Mongolians. Similar findings were obtained in association between number of household members and presence of DED. However, there is limited direct population-based evidence on this relationship, and the causal direction of the relationship is unclear. Future prospective studies should recruit a larger sample that is more representative of the population of China.

In this study, Schirmer's I test, TBUT, and OSDI scores were correlated with NEI-VFQ-25 scales. After controlling for factors, DED inversely associated with multiple subscales in NEI-VFQ-25 in Han and Mongolian adults. So far, only two large-sample size population-based study investigated the relationship between dry eye and quality of life. In a previous study involving 78,165 participants (19–94 years, 59.2% women) in Netherlands from 2006 to 2013, Morthen et al. found that dry eye is associated with low quality of life measured with SF-36 questionnaire. (11). In another study including 3,275 subjects in the United States from 2014, Paulsen et al. found that dry eye is also associated with low quality of life measured by both SF-36 and vision specific NEI-VFQ-25 questionnaires (13). Both these two previous studies were focused on dry eye symptoms (DES), which defined by a validated dry eye questionnaire, and a question “at least moderately bothersome symptoms present at minimum sometimes and/or treated with eye drops,” respectively. In China, a population-based cross-sectional study enrolled 229 subjects from Shanghai city focusing on vision-specific quality of life by NEI-VFQ-25 found that only two subscales, ocular pain and mental health, were related to DED (10). Another cross-sectional comparative study that enrolled 77 outpatients and 77 general participants with DED found that NEI-VFQ-25 composite score had a negative correlation with the OSDI score of all participants. The impairment of VR-QoL has a significant correlation with the severity of DES (26). However, in this study, associations were found between DED and all of the 12 NEI-VFQ subscales, although the effect was greatest on general vision. The effect of DED on all ethnicities in this investigation suggests that the impact of DED on the perception by an individual of their health is substantial and of importance as a public health problem.

This study has some strengths. Although the majority of correlations between DED and its factors were low power, such as age and female gender, to the best of our knowledge, this was the first large population-based study investigating epidemiology on DED among individuals from two ethnicities living in grasslands, which further enabled us to analyze the impacts of DED on VR-QOL. Therefore, our study added to the current knowledge of public health concern on preventive strategies for DED. Nevertheless, our study also has several limitations. First, this study had a cross-sectional design, and as such the causality of any findings between DED and factors as well as VR-QoL cannot be made. Second, in this study, we did not look at the epidemiology characteristics of DED severity. Hence, the diagnosis of DED was done based on both the presence of dry eye symptoms (OSDI) and clinical assessment (TBUT or Schirmer's I test), but did not include ocular surface staining, tear osmolarity, or meibomian dysfunction assessment because of the limited material conditions. In addition, TBUT was performed with topical anesthesia instead of balanced salt solution in this study. Third, causes of visual disturbances in the eligible participants with DED would have allowed this study to investigate if and to what extent reductions in VR-QoL were mediated by reductions in vision quality. Fourth, we did not collect information on menopausal states of the women, which may be associated with DED. Further studies are needed to identify this association among residents living in grasslands. Finally, when correcting for numerous comorbidities, we did not adjust for ocular disorders, such as diabetic retinopathy, age-related macular degeneration, retinal detachment, cataract, and glaucoma, because of the limited validity and reliability of self-report data, which might have overcorrected and, thus, underestimated the true effect of DED on VR-QoL.

In conclusion, both crude and adjusted prevalence of DED was relatively high in this study on Mongolian and Han populations living in the northern grasslands of China. Few studies have investigated DED in this area. Many similar risk factors previously found to be associated with DED were also found in this study and some novelties, such as anti-fatigue eye drop use, milk product intake, and number of household members, warrant further investigation. DED also proved to have a significant impact on VR-QoL, independent of other confounding factors. These findings on elder adults make DED prevention an important public health problem. Further longitudinal, multi-ethnical studies on DED in grasslands are necessary to improve broad representation, establish the causal relationship between factor exposure and onset of disease, and measure the impact of long-term disease on VR-QoL.

## Data Availability Statement

The raw data supporting the conclusions of this article will be made available by the authors, without undue reservation.

## Ethics Statement

The studies involving human participants were reviewed and approved by Inner Mongolia Chaoju Eye Hospital. The patients/participants provided their written informed consent to participate in this study.

## Author Contributions

All authors listed have made a substantial, direct, and intellectual contribution to the work and approved it for publication.

## Funding

The study was funded by the National Natural Science Foundation of China (Nos. 81300783 and 82003882), Liao Ning Revitalization Talents Program (No. XLYC1807082), Shenyang Young and Middle-aged Science and Technology Innovation Talent Support Program (Grant Number RC190146), Grassland Elite Project (No. CYYC10089), and Bethune Charitable Foundation (No. BJ-LM2019008J).

## Author Disclaimer

The content is solely the responsibility of the authors and does not necessarily represent the official views of the foundation.

## Conflict of Interest

The authors declare that the research was conducted in the absence of any commercial or financial relationships that could be construed as a potential conflict of interest.

## Publisher's Note

All claims expressed in this article are solely those of the authors and do not necessarily represent those of their affiliated organizations, or those of the publisher, the editors and the reviewers. Any product that may be evaluated in this article, or claim that may be made by its manufacturer, is not guaranteed or endorsed by the publisher.
