# Supplementary material for: Dry Eye Disease Among Mongolian and Han Older Adults in Grasslands of Northern China: Prevalence, Associated Factors, and Vision-Related Quality of Life
Source: Front Med (Lausanne). 2021 Nov 25;8:788545. doi: 10.3389/fmed.2021.788545 (PMC8655125; doi:10.3389/fmed.2021.788545)
Supplement: Supplementary file 1 [file Data_Sheet_1.docx]

**Supplementary files**

**Dry eye disease among Mongolian and Han older adults in grasslands of northern China: prevalence, associated factors and vision-related quality of life**

**Running title**: Epidemiology and VR-QoL on dry eye among Chinese Mongolian and Han

**Supplementary Figure 1. Study area and the sampling sites.**

**
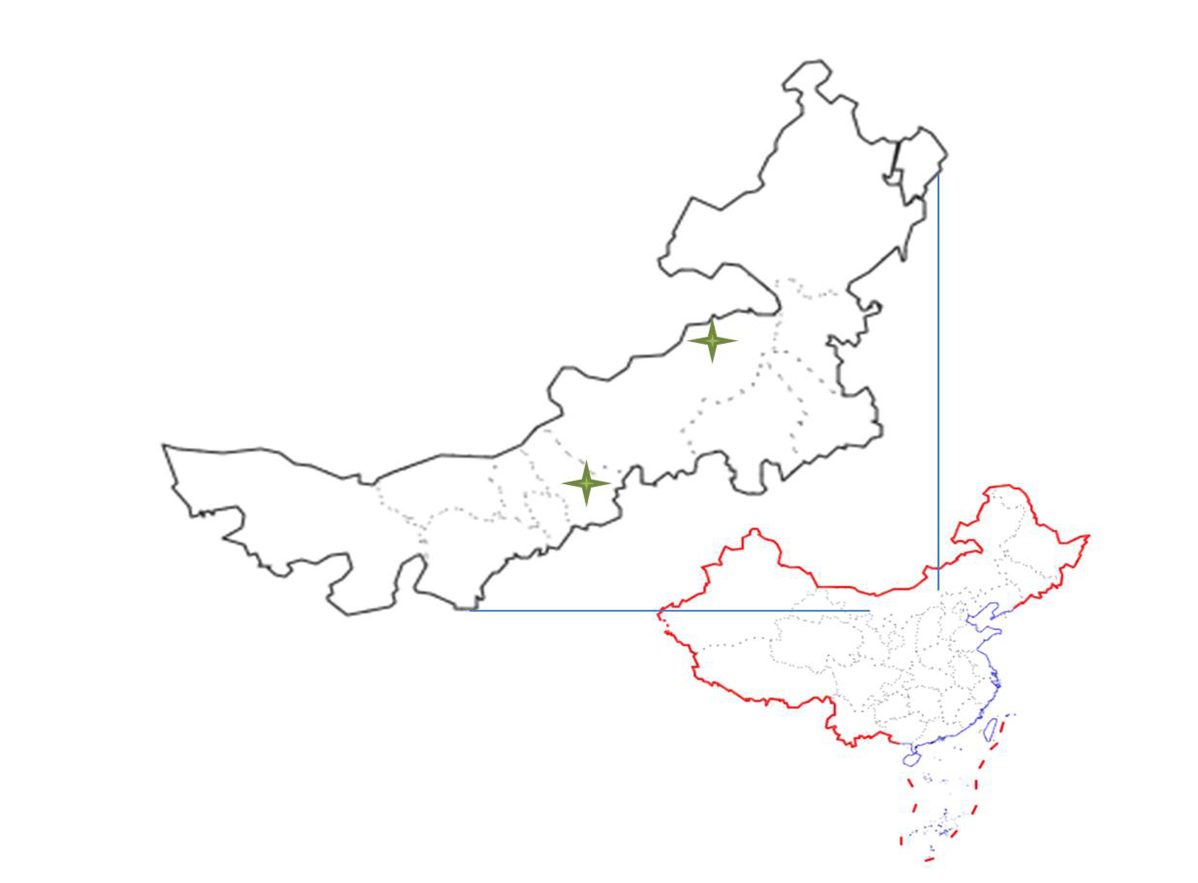
**

**Supplementary Table 1. Characteristics of Han participants**

|  | **N/Mean ± SD** | **Range** | **DED** | **No DED** | ***P*** |
| --- | --- | --- | --- | --- | --- |
| **Total** | 471 | - | 260(55.2%) | 211(44.8%) |  |
| **Residence** |  |  |  |  |  |
| Rural | 285 | - | 156(54.7%) | 129(45.3%) | 0.80 |
| Urban | 186 | - | 104(55.9%) | 82(44.1%) |  |
| **Sex** |  |  |  |  |  |
| Male | 140 | - | 63(45.0%) | 77(55.0%) | **< 0.01** |
| Female | 331 | - | 197(59.4%) | 134(40.6%) |  |
| **Occupation** |  |  |  |  |  |
| Worker | 17 | - | 9(52.9%) | 8(47.1%) | 0.59 |
| Farmer | 158 | - | 94(59.5%) | 64(40.5%) |  |
| Staff | 32 | - | 16(50.0%) | 16(50.0%) |  |
| Other | 264 | - | 141(53.4%) | 123(46.6%) |  |
| **Smoke** |  |  |  |  |  |
| Current | 59 | - | 31(52.5%) | 28(47.5%) | 0.56 |
| Never | 400 | - | 224(56.0%) | 176(44.0%) |  |
| Former | 12 | - | 5(41.7%) | 7(58.3%) |  |
| **Drink** |  |  |  |  |  |
| Current | 35 | - | 16(45.7%) | 19(54.3%) | 0.32 |
| Never | 421 | - | 236(56.1%) | 185(43.9%) |  |
| Former | 12 | - | 5(41.7%) | 7(58.3%) |  |
| **Level of education** |  |  |  |  |  |
| Primary school | 214 | - | 130(60.7%) | 84(39.3%) | **0.02** |
| Junior high school | 141 | - | 78(55.3%) | 63(44.7%) |  |
| Senior high school | 77 | - | 38(49.4%) | 39(50.6%) |  |
| College | 39 | - | 14(35.9%) | 25(64.1%) |  |
| **Diabetes** |  |  |  |  |  |
| With | 81 | - | 41(50.6%) | 40(49.4%) | 0.08 |
| Without | 371 | - | 203(54.7%) | 168(45.3%) |  |
| Unclear | 19 | - | 15(78.9%) | 4(21.1%) |  |
| **Hypertension** |  |  |  |  |  |
| With | 179 | - | 107(59.8%) | 72(40.2%) | **0.04** |
| Without | 278 | - | 142(51.1%) | 136(48.9%) |  |
| Unclear | 14 | - | 11(78.6%) | 3(21.4%) |  |
| **Anti-fatigue use** |  |  |  |  |  |
| Yes | 97 | - | 65(67.0%) | 32(33.0%) | **0.01** |
| Never | 374 | - | 195(52.0%) | 179(48.0%) |  |
| **Milk products intake** |  |  |  |  |  |
| Regular | 243 | - | 159(65.3%) | 84(34.7%) | **< 0.01** |
| Occasional | 228 | - | 102(44.9%) | 126(55.1%) |  |
| **Age (years)** | 62.59±9.82 | (22-88) | 63.88±9.75 | 61±9.69 | **< 0.01** |
| **Screen exposure per day (h)** | 2.68±1.79 | (0-12) | 2.63±1.84 | 2.74±1.73 | 0.49 |
| **Number of household members (n)** | 2.49±1.07 | (1-8) | 2.34±1.07 | 2.67±1.04 | **< 0.01** |
| **Annual household incomes (per ten thousand yuan)** | 3.89±3.82 | (0-20) | 3.76±3.63 | 4.04±4.04 | 0.42 |
| **Height (cm)** | 160.55±8.07 | (140-188) | 159.43±7.7 | 161.94±8.32 | **< 0.01** |
| **Weight (kg)** | 69.1±13.18 | (41-170) | 68.21±14.07 | 70.2±11.92 | 0.11 |
| **BMI** | 26.63±5.18 | (14.88-62.44) | 26.82±5.07 | 26.78±4.29 | 0.94 |
| **Height (cm)** | 95.75±12.18 | (39-129) | 96.01±12.26 | 95.42±12.11 | 0.60 |
| **Waist (cm)** | 84.52±12.65 | (35-116) | 84.7±12.22 | 84.29±13.19 | 0.73 |
| **SBP (mmHg)** | 139.79±31.49 | (85-190) | 141.04±39.9 | 138.26±15.97 | 0.34 |
| **DBP (mmHg)** | 81.1±13.1 | (50-144) | 80.13±13.93 | 82.3±11.93 | 0.07 |
| **Hart rate (per minute)** | 83.59±14.17 | (45-122) | 83.33±15.35 | 83.9±12.59 | 0.67 |
| **Schirmer's I test (mm)** | 7.41±6.69 | (0-30) | 5.52±5.13 | 9.74±7.6 | **< 0.01** |
| **TBUT (s)** | 5.81±4 | (0-14) | 3.78±2.49 | 8.31±4.09 | **< 0.01** |
| **OSDI score** | 25±15.62 | (0-100) | 30.57±13.98 | 18.13±14.82 | **< 0.01** |

DED: dry eye disease; BMI: body mass index; SBP: Systolic blood pressure; DBP: Diastolic blood pressure; TBUT: Tear film break up time; OSDI: Ocular surface disease index; SD standard deviation

**Supplementary Table 2. Characteristics of Mongolian participants**

|  | **N/Mean ± SD** | **Range** | **DED** | **No DED** | ***P*** |
| --- | --- | --- | --- | --- | --- |
| **Total** | 816 | - | 437(53.6%) | 379(46.4%) |  |
| **Residence** |  |  |  |  |  |
| Rural | 119 | - | 67(56.3%) | 52(43.7%) | 0.52 |
| Urban | 697 | - | 370(53.1%) | 327(46.9%) |  |
| **Sex** |  |  |  |  |  |
| Male | 292 | - | 153(52.4%) | 139(47.6%) | 0.60 |
| Female | 523 | - | 284(54.3%) | 239(45.7%) |  |
| **Occupation** |  |  |  |  |  |
| Worker | 5 | - | 1(20.0%) | 4(80.0%) | 0.44 |
| Farmer | 332 | - | 174(52.4%) | 158(47.6%) |  |
| Staff | 28 | - | 16(57.1%) | 12(42.9%) |  |
| Other | 448 | - | 244(54.5%) | 204(45.5%) |  |
| **Smoke** |  |  |  |  |  |
| Current | 139 | - | 80(57.6%) | 59(42.4%) | **0.02** |
| Never | 653 | - | 339(51.9%) | 314(48.1%) |  |
| Former | 21 | - | 17(81.0%) | 4(19.0%) |  |
| **Drink** |  |  |  |  |  |
| Current | 79 | - | 40(50.6%) | 39(49.4%) | 0.56 |
| Never | 712 | - | 380(53.4%) | 332(46.6%) |  |
| Former | 22 | - | 14(63.6%) | 8(36.4%) |  |
| **Level of education** |  |  |  |  |  |
| Primary school | 475 | - | 267(56.2%) | 208(43.8%) | 0.39 |
| Junior high school | 177 | - | 84(47.5%) | 93(52.5%) |  |
| Senior high school | 81 | - | 42(51.9%) | 39(48.1%) |  |
| College | 81 | - | 43(53.1%) | 38(46.9%) |  |
| Unclear | 2 | - | 1(50.0%) | 1(50.0%) |  |
| **Diabetes** |  |  |  |  |  |
| With | 56 | - | 31(55.4%) | 25(44.6%) | 0.81 |
| Without | 719 | - | 385(53.5%) | 334(46.5%) |  |
| Unclear | 35 | - | 17(48.6%) | 18(51.4%) |  |
| **Hypertension** |  |  |  |  |  |
| With | 295 | - | 169(57.3%) | 126(42.7%) | 0.15 |
| Without | 490 | - | 255(52.0%) | 235(48.0%) |  |
| Unclear | 29 | - | 12(41.4%) | 17(58.6%) |  |
| **Anti-fatigue eye-drop use** |  |  |  |  |  |
| Yes | 104 | - | 66(63.5%) | 38(36.5%) | **0.03** |
| No | 710 | - | 370(52.1%) | 340(47.9%) |  |
| **Milk products intake** |  |  |  |  |  |
| Regular | 784 | - | 417(53.2%) | 367(46.8%) | 0.38 |
| Occasional | 31 | - | 19(61.3%) | 12(38.7%) |  |
| **Age (years)** | 60.46±9.29 | (36-93) | 63.88±9.75 | 61±9.69 | **< 0.01** |
| **Screen exposure per day (h)** | 2.63±1.82 | (0-12) | 2.63±1.84 | 2.74±1.73 | 0.49 |
| **Number of household members (n)** | 2.63±1.28 | (1-11) | 2.34±1.07 | 2.67±1.04 | **< 0.01** |
| **Annual household incomes (per ten thousand yuan)** | 4.17±5.32 | (0-60) | 3.76±3.63 | 4.04±4.04 | 0.42 |
| **Height (cm)** | 160.35±8.63 | (103-185) | 159.43±7.7 | 161.94±8.32 | **< 0.01** |
| **Weight (kg)** | 71.52±14.95 | (41-140) | 68.21±14.07 | 70.2±11.92 | 0.11 |
| **BMI** | 27.76±5.51 | (16.02-66.92) | 26.82±5.07 | 26.78±4.29 | 0.94 |
| **Height (cm)** | 99.69±34.6 | (42-171) | 96.01±12.26 | 95.42±12.11 | 0.60 |
| **Waist (cm)** | 87.95±12.45 | (36-140) | 84.7±12.22 | 84.29±13.19 | 0.72 |
| **SBP (mmHg)** | 139.73±19.82 | (90-200) | 141.04±39.9 | 138.26±15.97 | 0.34 |
| **DBP (mmHg)** | 82.86±14.75 | (50-136) | 80.13±13.93 | 82.3±11.93 | 0.07 |
| **Hart rate (per minute)** | 80.11±13.25 | (42-119) | 83.33±15.35 | 83.9±12.59 | 0.67 |
| **Schirmer's I test (mm)** | 9.2±7.63 | (0-30) | 5.52±5.13 | 9.74±7.6 | **< 0.01** |
| **TBUT (s)** | 6.08±3.98 | (0-15) | 3.78±2.49 | 8.31±4.09 | **< 0.01** |
| **OSDI score** | 19.98±10.8 | (0-83.33) | 30.57±13.98 | 18.13±14.82 | **< 0.01** |

DED: dry eye disease; BMI: body mass index; SBP: Systolic blood pressure; DBP: Diastolic blood pressure; TBUT: Tear film break up time; OSDI: Ocular surface disease index; SD: standard deviation

**Supplementary Table 3. Logistic regression analysis of risk factors associated with definite DED among Han participants**

|  | **Crude** | | |  | **Adjusted*** | | |  | **Adjusted**** | | | |
| --- | --- | --- | --- | --- | --- | --- | --- | --- | --- | --- | --- | --- |
|  | OR | 95% CI | *P* |  | OR | 95% CI | *P* |  | OR | 95% CI | | *P* |
| **Age** | 1.03 | 1.01-1.05 | **< 0.01** |  | 1.03 | 1.01-1.05 | **< 0.01** |  | 1.02 | 1.00-1.04 | | **0.03** |
| **Sex (female)** | 1.79 | 1.20-2.66 | **< 0.01** |  | 1.75 | 1.17-2.62 | **0.01** |  | 1.79 | 1.19-2.70 | | **0.01** |
| **Residence (rural)** | 1.15 | 0.72-1.52 | 0.61 |  | 1.10 | 0.75-1.61 | 0.62 |  | 1.04 | 0.71-1.53 | | 0.84 |
| **Occupation** |  |  |  |  |  |  |  |  |  |  | |  |
| Worker | Ref |  |  |  | Ref |  |  |  | Ref |  | |  |
| Farmer | 1.31 | 0.48-3.56 | 0.60 |  | 1.01 | 0.36-2.83 | 0.99 |  | 0.80 | 0.28-2.28 | | 0.67 |
| Staff | 0.89 | 0.27-2.89 | 0.85 |  | 0.80 | 0.24-2.66 | 0.72 |  | 0.72 | 0.21-2.40 | | 0.59 |
| Other | 1.02 | 0.38-2.72 | 0.97 |  | 0.89 | 0.33-2.43 | 0.83 |  | 0.72 | 0.26-2.00 | | 0.53 |
| **Smoke** |  |  |  |  |  |  |  |  |  |  | |  |
| Current | Ref |  |  |  | Ref |  |  |  |  |  | |  |
| Never | 1.15 | 0.67-1.99 | 0.62 |  | 0.62 | 0.32-1.21 | 0.16 |  | 0.60 | 0.31-1.17 | | 0.14 |
| Former | 0.65 | 0.18-2.27 | 0.49 |  | 0.53 | 0.15-1.92 | 0.34 |  | 0.48 | 0.13-1.75 | | 0.27 |
| **Drink** |  |  |  |  |  |  |  |  |  |  | |  |
| Current | Ref |  |  |  | Ref |  |  |  | Ref |  | |  |
| Never | 1.52 | 0.76-3.03 | 0.24 |  | 0.86 | 0.39-1.88 | 0.70 |  | 0.80 | 0.36-1.76 | | 0.58 |
| Former | 0.85 | 0.23-3.20 | 0.81 |  | 0.72 | 0.19-2.77 | 0.64 |  | 0.68 | 0.18-2.60 | | 0.57 |
| **Screen exposure per day (h)** | 0.97 | 0.87-1.07 | 0.49 |  | 1.04 | 0.93-1.17 | 0.46 |  | 1.04 | 0.93-1.16 | | 0.51 |
| **Anti-fatigue eye-drop use (no)** | 0.53 | 0.33-0.85 | **0.01** |  | 0.51 | 0.32-0.83 | **0.01** |  | 0.50 | 0.31-0.81 | | **0.01** |
| **Milk products intake (regular)** | 0.43 | 0.30-0.63 | **< 0.01** |  | 0.41 | 0.28-0.60 | **< 0.01** |  | 0.43 | 0.29-0.63 | | **< 0.01** |
| **Education** |  |  |  |  |  |  |  |  |  |  | |  |
| Primary school | Ref |  |  |  | Ref |  |  |  | Ref |  | |  |
| Junior high school | 0.80 | 0.52-1.23 | 0.31 |  | 0.99 | 0.63-1.57 | 0.97 |  | 1.01 | 0.63-1.60 | | 0.98 |
| Senior high school | 0.63 | 0.37-1.06 | 0.08 |  | 0.84 | 0.48-1.47 | 0.54 |  | 0.83 | 0.48-1.46 | | 0.52 |
| College | 0.36 | 0.18-0.74 | **0.01** |  | 0.57 | 0.26-1.23 | 0.15 |  | 0.59 | 0.27-1.27 | | 0.18 |
| **Number of household members (n)** | 0.74 | 0.62-0.89 | **< 0.01** |  | 0.77 | 0.64-0.93 | **0.01** |  | 0.77 | 0.64-0.93 | | **0.01** |
| **Annual household incomes (per ten thousand yuan)** | 0.98 | 0.94-1.03 | 0.42 |  | 1.02 | 0.96-1.07 | 0.58 |  | 1.03 | 0.97-1.08 | | 0.33 |
| **Diabetes** |  |  |  |  |  |  |  |  |  |  | |  |
| With | Ref |  |  |  | Ref |  |  |  | Ref |  | |  |
| Without | 1.18 | 0.73-1.91 | 0.50 |  | 1.27 | 0.77-2.07 | 0.35 |  | 1.31 | 0.80-2.16 | | 0.28 |
| Unclear | 3.66 | 1.12-11.98 | **0.03** |  | 3.81 | 1.12-12.92 | **0.03** |  | 3.51 | 1.03-11.99 | | 0.05 |
| **Hypertension** |  |  |  |  |  |  |  |  |  | |  |  |
| With | Ref |  |  |  | Ref |  |  |  | Ref | |  |  |
| Without | 0.70 | 0.48-1.03 | 0.07 |  | 0.85 | 0.57-1.26 | 0.42 |  | 0.86 | | 0.57-1.28 | 0.46 |
| Unclear | 2.47 | 0.67-9.15 | 0.18 |  | 2.79 | 0.73-10.60 | 0.13 |  | 2.65 | | 0.70-10.07 | 0.15 |
| **Height (cm)** | 0.96 | 0.94-0.98 | **< 0.01** |  | 0.99 | 0.96-1.02 | 0.33 |  | 0.99 | | 0.96-1.02 | 0.45 |
| **Weight (kg)** | 0.99 | 0.98-1.00 | 0.11 |  | 1.00 | 0.98-1.01 | 0.54 |  | 1.00 | | 0.98-1.01 | 0.72 |
| **BMI** | 1.01 | 0.97-1.04 | 0.70 |  | 1.00 | 0.96-1.04 | 0.92 |  | 1.00 | | 0.97-1.04 | 0.91 |
| **Height (cm)** | 1.00 | 0.99-1.02 | 0.60 |  | 1.00 | 0.99-1.02 | 0.62 |  | 1.01 | | 0.99-1.02 | 0.52 |
| **Waist (cm)** | 1.00 | 0.99-1.02 | 0.73 |  | 1.00 | 0.99-1.02 | 0.86 |  | 1.00 | | 0.99-1.02 | 0.86 |
| **SBP (mmHg)** | 1.00 | 1.00-1.01 | 0.38 |  | 1.00 | 1.00-1.01 | 0.73 |  | 1.00 | | 1.00-1.09 | 0.64 |
| **DBP (mmHg)** | 0.99 | 0.97-1.00 | 0.08 |  | 0.99 | 0.97-1.00 | 0.08 |  | 0.99 | | 0.97-1.00 | 0.11 |
| **Hart rate (per minute)** | 1.00 | 0.98-1.01 | 0.67 |  | 0.99 | 0.98-1.09 | 0.41 |  | 0.99 | | 0.98-1.01 | 0.32 |
| **Schirmer's I test (mm)** | 0.90 | 0.87-0.93 | **< 0.01** |  | 0.90 | 0.87-0.93 | **< 0.01** |  | 0.90 | | 0.87-0.93 | **< 0.01** |
| **TBUT (s)** | 0.71 | 0.66-0.75 | **< 0.01** |  | 0.71 | 0.66-0.76 | **< 0.01** |  | 0.70 | | 0.66-0.75 | **< 0.01** |
| **OSDI score** | 1.07 | 1.05-1.09 | **< 0.01** |  | 1.07 | 1.05-1.09 | **< 0.01** |  | 1.07 | | 1.05-1.09 | **< 0.01** |

DED: dry eye disease; BMI: body mass index; SBP: Systolic blood pressure; DBP: Diastolic blood pressure; TBUT: Tear film break up time; OSDI: Ocular surface disease index; OR: odds ratio; CI: confidence interval.

* adjusted with age, sex, and ethnic

** adjusted with age, sex, ethnic, and number of household members

**Supplementary Table 4. Logistic regression analysis of risk factors associated with definite DED among Mongolian participants**

|  | **Crude** | | |  | **Adjusted*** | | |  | **Adjusted**** | | |
| --- | --- | --- | --- | --- | --- | --- | --- | --- | --- | --- | --- |
|  | OR | 95% CI | *P* |  | OR | 95% CI | *P* |  | OR | 95% CI | *P* |
| **Age** | 1.04 | 1.03-1.06 | **< 0.01** |  | 1.04 | 1.03-1.06 | **< 0.01** |  | 1.04 | 1.02-1.05 | **< 0.01** |
| **Sex (female)** | 1.08 | 0.81-1.44 | 0.60 |  | 1.12 | 0.84-1.50 | 0.44 |  | 1.13 | 0.84-1.52 | 0.41 |
| **Residence (rural)** | 1.47 | 0.59-1.30 | 0.31 |  | 0.84 | 0.56-1.25 | 0.39 |  | 0.80 | 0.53-1.19 | 0.27 |
| **Occupation** |  |  |  |  |  |  |  |  |  |  |  |
| Worker | Ref |  |  |  | Ref |  |  |  | Ref |  |  |
| Farmer | 4.41 | 0.49-39.83 | 0.19 |  | 4.35 | 0.47-40.13 | 0.20 |  | 4.21 | 0.45-39.07 | 0.21 |
| Staff | 5.33 | 0.53-54.03 | 0.16 |  | 5.64 | 0.54-58.62 | 0.15 |  | 5.38 | 0.51-56.34 | 0.16 |
| Other | 4.78 | 0.53-43.14 | 0.16 |  | 4.78 | 0.52-43.92 | 0.17 |  | 4.39 | 0.48-40.67 | 0.19 |
| **Smoke** |  |  |  |  |  |  |  |  |  |  |  |
| Current | Ref |  |  |  | Ref |  |  |  | Ref |  |  |
| Never | 0.80 | 0.55-1.15 | 0.23 |  | 0.70 | 0.47-1.04 | 0.08 |  | 0.71 | 0.47-1.05 | 0.09 |
| Former | 3.13 | 1.00-9.80 | 0.05 |  | 2.66 | 0.83-8.50 | 0.10 |  | 2.85 | 0.89-9.13 | 0.08 |
| **Drink** |  |  |  |  |  |  |  |  |  |  |  |
| Current | Ref |  |  |  | Ref |  |  |  | Ref |  |  |
| Never | 1.12 | 0.70-1.78 | 0.64 |  | 0.92 | 0.56-1.52 | 0.75 |  | 0.95 | 0.57-1.58 | 0.84 |
| Former | 1.71 | 0.64-4.52 | 0.28 |  | 1.81 | 0.65-5.06 | 0.26 |  | 1.80 | 0.64-5.03 | 0.26 |
| **Screen exposure per day (h)** | 0.98 | 0.91-1.06 | 0.57 |  | 1.04 | 0.96-1.13 | 0.33 |  | 1.03 | 0.95-1.12 | 0.43 |
| **Anti-fatigue eye-drop use (no)** | 0.63 | 0.41-0.96 | **0.03** |  | 0.61 | 0.40-0.94 | **0.02** |  | 0.62 | 0.40-0.96 | **0.03** |
| **Milk products intake (regular)** | 1.39 | 0.67-2.91 | 0.38 |  | 1.52 | 0.72-3.20 | 0.28 |  | 1.43 | 0.67-3.03 | 0.36 |
| **Education** |  |  |  |  |  |  |  |  |  |  |  |
| Primary school | Ref |  |  |  | Ref |  |  |  | Ref |  |  |
| Junior high school | 0.70 | 0.50-1.00 | 0.05 |  | 0.77 | 0.54-1.10 | 0.15 |  | 0.81 | 0.57-1.16 | 0.26 |
| Senior high school | 0.84 | 0.52-1.35 | 0.47 |  | 1.00 | 0.62-1.63 | 0.99 |  | 1.01 | 0.62-1.64 | 0.98 |
| College | 0.88 | 0.55-1.41 | 0.60 |  | 1.11 | 0.68-1.82 | 0.67 |  | 1.11 | 0.68-1.82 | 0.68 |
| Other | 0.78 | 0.05-12.53 | 0.86 |  | 0.86 | 0.05-14.18 | 0.92 |  | 0.75 | 0.05-12.24 | 0.84 |
| **Number of household members (n)** | 0.76 | 0.68-0.86 | **< 0.01** |  | 0.80 | 0.72-0.90 | **< 0.01** |  | 0.80 | 0.72-0.90 | **< 0.01** |
| **Annual household incomes (per ten thousand yuan)** | 0.97 | 0.94-1.00 | **0.03** |  | 0.98 | 0.96-1.01 | 0.27 |  | 0.99 | 0.96-1.02 | 0.55 |
| **Diabetes** |  |  |  |  |  |  |  |  |  |  |  |
| With | Ref |  |  |  | Ref |  |  |  | Ref |  |  |
| Without | 0.93 | 0.54-1.61 | 0.79 |  | 0.98 | 0.56-1.71 | 0.94 |  | 0.97 | 0.55-1.70 | 0.91 |
| Unclear | 0.76 | 0.33-1.78 | 0.53 |  | 0.84 | 0.35-1.99 | 0.69 |  | 0.81 | 0.34-1.94 | 0.64 |
| **Hypertension** |  |  |  |  |  |  |  |  |  |  |  |
| With | Ref |  |  |  | Ref |  |  |  | Ref |  |  |
| Without | 0.81 | 0.61-1.08 | 0.15 |  | 0.95 | 0.70-1.29 | 0.75 |  | 1.00 | 0.73-1.35 | 0.97 |
| Unclear | 0.53 | 0.24-1.14 | 0.10 |  | 0.52 | 0.24-1.15 | 0.11 |  | 0.52 | 0.23-1.16 | 0.11 |
| **Height (cm)** | 0.98 | 0.96-1.00 | **0.01** |  | 0.99 | 0.97-1.01 | 0.23 |  | 0.99 | 0.97-1.01 | 0.27 |
| **Weight (kg)** | 1.00 | 0.99-1.01 | 0.82 |  | 1.00 | 0.99-1.01 | 0.94 |  | 1.00 | 0.99-1.01 | 0.87 |
| **BMI** | 1.02 | 0.99-1.04 | 0.18 |  | 1.01 | 0.99-1.04 | 0.43 |  | 1.01 | 0.99-1.04 | 0.41 |
| **Height (cm)** | 1.00 | 1.00-1.01 | 0.36 |  | 1.00 | 1.00-1.01 | 0.43 |  | 1.00 | 1.00-1.01 | 0.46 |
| **Waist (cm)** | 1.01 | 0.99-1.02 | 0.41 |  | 1.00 | 0.99-1.01 | 0.70 |  | 1.00 | 0.99-1.01 | 0.71 |
| **SBP (mmHg)** | 1.00 | 0.99-1.01 | 0.96 |  | 0.99 | 0.99-1.00 | 0.09 |  | 0.99 | 0.99-1.00 | 0.08 |
| **DBP (mmHg)** | 1.00 | 0.99-1.01 | 0.82 |  | 1.00 | 0.99-1.01 | 0.96 |  | 1.00 | 0.99-1.01 | 0.94 |
| **Hart rate (per minute)** | 1.00 | 0.99-1.01 | 0.89 |  | 1.00 | 0.99-1.01 | 0.63 |  | 1.00 | 0.99-1.01 | 0.61 |
| **Schirmer's I test (mm)** | 0.90 | 0.88-0.92 | **< 0.01** |  | 0.90 | 0.88-0.92 | **< 0.01** |  | 0.90 | 0.87-0.92 | **< 0.01** |
| **TBUT (s)** | 0.69 | 0.66-0.73 | **< 0.01** |  | 0.69 | 0.66-0.73 | **< 0.01** |  | 0.69 | 0.66-0.73 | **< 0.01** |
| **OSDI score** | 1.14 | 1.11-1.16 | **< 0.01** |  | 1.13 | 1.11-1.16 | **< 0.01** |  | 1.13 | 1.11-1.15 | **< 0.01** |

DED: dry eye disease; BMI: body mass index; SBP: Systolic blood pressure; DBP: Diastolic blood pressure; TBUT: Tear film break up time; OSDI: Ocular surface disease index; OR: odds ratio; CI: confidence interval.

* adjusted with age, sex, and ethnic

** adjusted with age, sex, ethnic, and number of household members

**Supplement Table 5. Correlation between DED subscale and NEI VFQ-25 subscale among Han participants**

|  | **Overall** | **General health** | **General vision** | **Ocular pain** | **Near activities** | **Distance activities** | **Social functioning** | **Mental health** | **Role difficulties** | **Dependency** | **Driving** | **Color vision** | **Peripheral vision** |
| --- | --- | --- | --- | --- | --- | --- | --- | --- | --- | --- | --- | --- | --- |
| **Schirmer's I test (mm)** | -0.01 | -0.05 | 0.04 | -0.05 | 0.00 | 0.07 | 0.05 | -0.06 | -0.03 | 0.04 | 0.02 | -0.03 | -0.01 |
| **TBUT (s)** | 0.05 | 0.06 | 0.07 | -0.09 | 0.03 | 0.04 | 0.07 | 0.01 | 0.03 | 0.00 | 0.11 | -0.03 | 0.01 |
| **OSDI score** | -0.56** | -0.30** | -0.50** | 0.20** | -0.24** | -0.29** | -0.27** | -0.09* | -0.24** | -0.53** | -0.52** | -0.13** | -0.24** |

DED: Dry eye disease; TBUT: Tear film break up time; OSDI: Ocular surface disease index; NEI VFQ-25: 25-item National Eye Institute Visual Functioning Questionnaire.

* *P* < 0.05; ** *P* < 0.01

**Supplement Table 6. Correlation between DED subscale and NEI VFQ-25 subscale among Mongolian participants**

|  | **Overall** | **General health** | **General vision** | **Ocular pain** | **Near activities** | **Distance activities** | **Social functioning** | **Mental health** | **Role difficulties** | **Dependency** | **Driving** | **Color vision** | **Peripheral vision** |
| --- | --- | --- | --- | --- | --- | --- | --- | --- | --- | --- | --- | --- | --- |
| **Schirmer's I test (mm)** | -0.03 | -0.08* | -0.00 | 0.00 | 0.00 | -0.02 | -0.02 | -0.01 | 0.03 | 0.03 | -0.05 | 0.04 | -0.01 |
| **TBUT (s)** | 0.07 | 0.04 | 0.08* | 0.03 | 0.02 | 0.04 | -0.01 | -0.03 | 0.07 | 0.00 | 0.10 | 0.03 | 0.05 |
| **OSDI score** | -0.39** | -0.02 | -0.32** | 0.11** | -0.19** | -0.22** | -0.19** | -0.14** | -0.18** | -0.45** | -0.57** | -0.10** | -0.18** |

DED: Dry eye disease; TBUT: Tear film break up time; OSDI: Ocular surface disease index; NEI VFQ-25: NEI VFQ-25: 25-item National Eye Institute Visual Functioning Questionnaire.

* *P* < 0.05; ** *P* < 0.01

**Supplementary Table 7. Multiple linear regression on dry eye disease and vision-related quality of life based on NEI-VFQ-25 among Han participants**

|  | **DED** | | | |  | **OSDI score** | | | |  | **Schirmer's I test (mm)** | | | |  | **TBUT (s)** | | | |
| --- | --- | --- | --- | --- | --- | --- | --- | --- | --- | --- | --- | --- | --- | --- | --- | --- | --- | --- | --- |
|  |  |  | **95% CI** | |  |  |  | **95% CI** | |  |  |  | **95% CI** | |  |  |  | **95% CI** | |
|  | ***β**** | ***P*** | **low** | **Up** |  | ***β**** | ***P*** | **low** | **up** |  | ***β**** | ***P*** | **low** | **up** |  | ***β**** | ***P*** | **low** | **up** |
| **Overall** | -0.18 | **< 0.01** | -0.27 | -0.08 |  | -0.49 | **< 0.01** | -0.56 | -0.43 |  | -0.06 | 0.27 | -0.16 | 0.05 |  | 0.02 | 0.74 | -0.08 | 0.11 |
| **General health** | -0.11 | **0.02** | -0.20 | -0.02 |  | -0.20 | **< 0.01** | -0.28 | -0.13 |  | -0.14 | **0.01** | -0.24 | -0.04 |  | 0.04 | 0.36 | -0.05 | 0.13 |
| **General vision** | -0.21 | **< 0.01** | -0.31 | -0.11 |  | -0.42 | **< 0.01** | -0.49 | -0.34 |  | 0.02 | 0.78 | -0.09 | 0.12 |  | 0.06 | 0.27 | -0.04 | 0.15 |
| **Ocular pain** | 0.02 | 0.71 | -0.10 | 0.14 |  | 0.18 | **< 0.01** | 0.09 | 0.28 |  | -0.03 | 0.69 | -0.15 | 0.10 |  | -0.04 | 0.52 | -0.15 | 0.08 |
| **Near activities** | -0.12 | **0.02** | -0.21 | -0.02 |  | -0.26 | **< 0.01** | -0.33 | -0.18 |  | -0.01 | 0.89 | -0.11 | 0.10 |  | < 0.01 | 0.93 | -0.10 | 0.09 |
| **Distance activities** | -0.13 | **0.02** | -0.23 | -0.02 |  | -0.28 | **< 0.01** | -0.36 | -0.20 |  | 0.05 | 0.40 | -0.06 | 0.16 |  | 0.03 | 0.57 | -0.07 | 0.13 |
| **Social functioning** | -0.12 | **0.01** | -0.22 | -0.03 |  | -0.23 | **< 0.01** | -0.30 | -0.15 |  | 0.04 | 0.39 | -0.06 | 0.15 |  | 0.06 | 0.18 | -0.03 | 0.16 |
| **Mental health** | 0.08 | 0.19 | -0.04 | 0.20 |  | -0.13 | **0.01** | -0.23 | -0.03 |  | -0.12 | 0.07 | -0.25 | 0.01 |  | -0.07 | 0.27 | -0.18 | 0.05 |
| **Role difficulties** | -0.03 | 0.56 | -0.15 | 0.08 |  | -0.22 | **< 0.01** | -0.31 | -0.12 |  | -0.04 | 0.56 | -0.16 | 0.09 |  | -0.01 | 0.91 | -0.12 | 0.11 |
| **Dependency** | -0.19 | **< 0.01** | -0.28 | -0.10 |  | -0.43 | **< 0.01** | -0.50 | -0.37 |  | < 0.01 | 0.94 | -0.10 | 0.11 |  | -0.01 | 0.90 | -0.10 | 0.09 |
| **Driving** | -0.06 | 0.42 | -0.20 | 0.08 |  | -0.17 | **< 0.01** | -0.27 | -0.07 |  | -0.05 | 0.53 | -0.19 | 0.10 |  | -0.03 | 0.72 | -0.16 | 0.11 |
| **Color vision** | 0.06 | 0.18 | -0.03 | 0.15 |  | -0.15 | **< 0.01** | -0.22 | -0.08 |  | -0.04 | 0.39 | -0.13 | 0.05 |  | -0.05 | 0.27 | -0.13 | 0.04 |
| **Peripheral vision** | 0.03 | 0.49 | -0.06 | 0.12 |  | -0.18 | **< 0.01** | -0.26 | -0.11 |  | -0.07 | 0.19 | -0.16 | 0.03 |  | -0.05 | 0.32 | -0.13 | 0.04 |

TBUT: Tear film break up time; OSDI: Ocular surface disease index; CI: Confidence interval; DED: Dry eye disease; NEI-VFQ-25: 25-item National Eye Institute Visual Functioning Questionnaire.

* Adjusted with age, sex, annual household incomes, and diabetes.

**Supplementary Table 8. Multiple linear regression on dry eye disease and vision-related quality of life based on NEI-VFQ-25 among Mongolian participants**

|  | **DED** | | | |  | **OSDI score** | | | |  | **Schirmer's I test (mm)** | | |  |  |  | **TBUT (s)** | | | |
| --- | --- | --- | --- | --- | --- | --- | --- | --- | --- | --- | --- | --- | --- | --- | --- | --- | --- | --- | --- | --- |
|  |  |  | **95% CI** | |  |  |  | **95% CI** | |  |  |  | **95% CI** | |  |  |  |  | **95% CI** | |
|  | ***β**** | ***P*** | **low** | **up** |  | ***β**** | ***P*** | **low** | **up** |  | ***β**** | ***P*** | **low** | **up** |  |  | ***β**** | ***P*** | **low** | **up** |
| **Overall** | -0.12 | **< 0.01** | -0.18 | -0.06 |  | -0.49 | **< 0.01** | -0.56 | -0.43 |  | 0.02 | 0.50 | -0.04 | 0.08 |  |  | 0.03 | 0.41 | -0.04 | 0.09 |
| **General health** | -0.03 | 0.33 | -0.10 | 0.03 |  | 0.01 | 0.77 | -0.07 | 0.09 |  | -0.02 | 0.44 | -0.08 | 0.04 |  |  | 0.02 | 0.57 | -0.05 | 0.08 |
| **General vision** | -0.17 | **< 0.01** | -0.23 | -0.11 |  | -0.36 | **< 0.01** | -0.43 | -0.29 |  | 0.04 | 0.21 | -0.02 | 0.10 |  |  | 0.04 | 0.16 | -0.02 | 0.10 |
| **Ocular pain** | -0.04 | 0.13 | -0.10 | 0.01 |  | 0.11 | **< 0.01** | 0.05 | 0.18 |  | 0.02 | 0.44 | -0.03 | 0.07 |  |  | 0.05 | 0.11 | -0.01 | 0.10 |
| **Near activities** | -0.05 | 0.19 | -0.11 | 0.02 |  | -0.20 | **< 0.01** | -0.28 | -0.12 |  | 0.01 | 0.81 | -0.06 | 0.07 |  |  | < 0.01 | 0.90 | -0.07 | 0.06 |
| **Distance activities** | -0.07 | **0.04** | -0.13 | -0.01 |  | -0.28 | **< 0.01** | -0.35 | -0.21 |  | -0.01 | 0.83 | -0.07 | 0.05 |  |  | 0.01 | 0.80 | -0.05 | 0.07 |
| **Social functioning** | -0.04 | 0.26 | -0.11 | 0.03 |  | -0.43 | **< 0.01** | -0.50 | -0.35 |  | -0.04 | 0.24 | -0.11 | 0.03 |  |  | -0.01 | 0.77 | -0.08 | 0.06 |
| **Mental health** | 0.03 | 0.29 | -0.02 | 0.08 |  | -0.13 | **< 0.01** | -0.20 | -0.07 |  | 0.01 | 0.81 | -0.04 | 0.06 |  |  | -0.02 | 0.40 | -0.08 | 0.03 |
| **Role difficulties** | -0.07 | **0.02** | -0.13 | -0.01 |  | -0.25 | **< 0.01** | -0.31 | -0.18 |  | 0.03 | 0.30 | -0.03 | 0.08 |  |  | 0.03 | 0.31 | -0.03 | 0.09 |
| **Dependency** | -0.09 | **0.01** | -0.16 | -0.02 |  | -0.48 | **< 0.01** | -0.56 | -0.41 |  | 0.06 | 0.07 | < 0.01 | 0.13 |  |  | 0.01 | 0.76 | -0.06 | 0.08 |
| **Driving** | -0.12 | **0.01** | -0.22 | -0.03 |  | -0.30 | **< 0.01** | -0.39 | -0.22 |  | -0.01 | 0.89 | -0.10 | 0.08 |  |  | 0.01 | 0.81 | -0.08 | 0.10 |
| **Color vision** | -0.05 | 0.17 | -0.13 | 0.02 |  | -0.28 | **< 0.01** | -0.37 | -0.20 |  | 0.04 | 0.27 | -0.03 | 0.11 |  |  | 0.04 | 0.29 | -0.03 | 0.11 |
| **Peripheral vision** | -0.08 | 0.03 | -0.15 | -0.01 |  | -0.35 | **< 0.01** | -0.44 | -0.27 |  | -0.02 | 0.59 | -0.09 | 0.05 |  |  | 0.03 | 0.42 | -0.04 | 0.10 |

TBUT: Tear film break up time; OSDI: Ocular surface disease index; CI: Confidence interval; DED: Dry eye disease; NEI-VFQ-25: 25-item National Eye Institute Visual Functioning Questionnaire.

* Adjusted with age, sex, annual household incomes, and diabetes.
